# Bedtime tetrahydrocannabinol ingestion reduces morning orthostatic cardiovascular reactivity in adults: An exploratory study

**DOI:** 10.14814/phy2.71076

**Published:** 2026-08-28

**Authors:** Joshua E. Gonzalez, Alicia V. Stewart, LaTroy D. Robinson, Noal A. Clemons, Omar H. Ordaz, Jonathan S. Emens, Saurabh S. Thosar, Steven A. Shea, Nicole P. Bowles

**Affiliations:** ^1^ Oregon Institute of Occupational Health Sciences Oregon Health & Science University Portland Oregon USA; ^2^ College of Nursing and Health Sciences Texas A&M University‐Corpus Christi Corpus Christi Texas USA; ^3^ VA Portland Healthcare System Portland Oregon USA; ^4^ Department of Psychiatry Oregon Health & Science University Portland Oregon USA; ^5^ Knight Cardiovascular Institute, School of Medicine Oregon Health & Science University Portland Oregon USA; ^6^ School of Nursing Oregon Health & Science University Portland Oregon USA; ^7^ Oregon Health and Science University‐Portland State University School of Public Health Portland Oregon USA

**Keywords:** autonomic function, baroreflex, hemodynamics, marijuana, physiological tolerance, postural stress

## Abstract

Acute cannabis use and its primary psychoactive constituent tetrahydrocannabinol (THC) can induce postural dizziness, indicating increased risk of orthostatic instability. However, it is unknown if vulnerabilities persist the morning after bedtime THC consumption. This study investigated the influence of bedtime THC consumption on cardiovascular variables during an orthostatic challenge. Nine individuals with no cannabis use and eight individuals who regularly use cannabis participated in a tilt table test associated with a 3‐day in‐laboratory stay. Participants underwent an acclimatization day, followed by a placebo dosing day, and a 10 mg THC dosing day. Placebo and THC pills were given 1 h before a participant's habitual bedtime, and the tilt table test was performed ~1 h upon awakening. Participants were instrumented with an electrocardiogram and an automated sphygmomanometer. Fluid intake and output were measured throughout the study. The morning after THC administration, individuals with no cannabis use reported a higher frequency of dizziness and exhibited a significant reduction in both diastolic blood pressure and heart rate reactivity to tilt (reactivity = tilt ‐ baseline) compared to the morning after placebo. Our results indicate a potential increased risk for orthostatic instability the morning after cannabis consumption in individuals with no cannabis use history.

## 1 INTRODUCTION

Tetrahydrocannabinol (THC) is the most abundant cannabinoid within most cannabis plant cultivars and is responsible for the euphoric properties of cannabis (Rock & Parker, 2021). Despite the increased perception of cannabis as a low‐risk product (Levy et al., 2021), several epidemiological studies have demonstrated an association between cannabis use and an increased risk of adverse cardiovascular outcomes, including myocardial infarction and stroke (Jeffers et al., 2024; Ladha et al., 2021; Mittleman et al., 2001; van Amsterdam & van den Brink, 2024). Dizziness and lightheadedness are commonly reported symptoms of cannabis use (Robson, 2001). Studies have reported that postural dizziness and orthostatic hypotension can occur when conducting a brief stand test immediately after THC infusions or cannabis smoking (Mathew et al., 2003; Merritta et al., 2009). Acute cannabis use reduces cardiac baroreflex sensitivity (Nardone et al., 2023), which is a crucial mechanism for maintaining stable blood pressure during postural change. The cardiac baroreflex plays an essential role in preventing dizziness and/or syncope during an orthostatic challenge. However, whether residual impairments in orthostatic tolerance or autonomic cardiovascular control persist beyond the acute intoxication window, particularly among individuals that regularly use cannabis compared to those with no cannabis use, remains unclear. With the rapid adoption of cannabis and THC‐containing products as sleep aids, it is particularly relevant to examine the residual cardiovascular effects that may continue into the morning hours in individuals who regularly use cannabis as well as individuals with no cannabis use. Therefore, we hypothesize that individuals with no use of cannabis will exhibit enhanced symptomatology and reduced cardiovascular reactivity during an orthostatic challenge. Therefore, the purpose of this ancillary analysis of a parent study (Gonzalez et al., 2026) was to investigate the impact of bedtime THC ingestion on the subsequent morning's cardiovascular responses to an orthostatic stress.

## 2 METHODS

### 2.1 Study design

This repeated measures, single blinded, placebo controlled study was conducted in the Oregon Clinical & Translational Research Institute Laboratory at Oregon Health & Science University. Participants were admitted for a 3‐night stay consisting of an acclimatization night, a placebo dosing night, and a THC dosing night using 10 mg of dronabinol (Bajtel et al., 2022). The order of administration was chosen to avoid the potential impact of any drug carry over effects, as it is known that Δ^9^‐THC and its metabolites are present in plasma for hours to days after use (Musshoff & Madea, 2006). Upon admission (acclimatization day), participants were instrumented with ECG and introduced to all tasks they would be asked to perform during their stay, including the tilt table test. The placebo and 10 mg THC tablet were administered 1 h before participants' habitual sleep time. Upon awakening, participants remained in bed in a supine position for ~20 min and then after a further ~20 min (to allow participants to use the bathroom), they lay supine on a tilt table, secured in position with comfortable Velcro straps, and then instrumented with a brachial automated oscillometric sphygmomanometer. The tilt‐table procedure consisted of a 20‐min resting supine baseline (starting 40 min after awakening), a 30‐min passive 80° head‐up tilt test, and a subsequent 20‐min supine recovery. Participants were instructed to minimize the use of their leg muscles during the tilting protocol to minimize the contribution to skeletal muscle contractions on venous return to the heart.

### 2.2 Participants

The participants in this exploratory study are the same cohort described in the parent study, where the full set of participant characteristics and a consort flow diagram are reported in detail (Gonzalez et al., 2026). This exploratory study included nine individuals with no cannabis use in the past year, fewer than 10 lifetime cannabis exposures, and a urine toxicology screen negative for THC metabolites. Data are presented as mean ± SD (6 female, 5 Non‐Hispanic White and 4 Asian, age = 26.3 ± 3.5 years, BMI = 22.9 ± 3.3 kg/m^2^). We recruited eight individuals who use cannabis regularly identified as >3 uses per week for at least 3 months and with a positive urine toxicology screen for THC metabolites (4 female, 4 Non‐Hispanic White, 2 Asian,1 Hispanic, 1 declined to answer, age = 24.1 ± 3.1 years, BMI = 24 ± 3.1 kg/m^2^, Cannabis Use Disorder Identification Test Revised [CUDIT‐R] Score = 8 ± 1). One individual who regularly used cannabis did not consent to do the tilt table test but completed the parent study (Gonzalez et al., 2026). Individuals who regularly use cannabis were eligible if they reported frequent cannabis use but did not meet study thresholds suggestive of cannabis use disorder or clinically meaningful withdrawal symptoms (CUDIT‐R, <12 and <2 symptoms on the Marijuana Withdrawal Checklist [MWC]) (Adamson et al., 2010; Budney et al., 1999). The primary purpose of the parent study was to investigate the influence of THC ingestion before bedtime on sleep parameters (Gonzalez et al., 2026). The participants were screened for obesity (body mass index), diabetes (fasting glucose levels), general health status (complete blood count), and pregnancy (confirmed with urine test). Apart from cannabis, participants were drug‐free (including all caffeine sources, nicotine, alcohol, and herbal medications) for the duration of the screening and study period (at least 1 week prior) as confirmed by urine tests (Drugsmart 12‐panel cup; Speares Medical and NicAlert; Nymox). Cannabis use frequency among the individuals who regularly use cannabis was determined using the time line follow back (Hjorthøj et al., 2012) and confirmed with urinalysis. Participants maintained a consistent sleep–wake cycle for at least 1 week leading up to laboratory admission to stabilize sleep–wake cycles and the internal body clock in line with the aims of the parent study. During this time individuals who regularly used cannabis were allowed to use cannabis ad libitum but were asked to abstain 3 days before laboratory admission. Among individuals who regularly use cannabis, the most common route of administration was smoking cannabis using bowls or bongs (*n* = 6), followed by vaporized THC (*n* = 1) and joints (*n* = 1). During the 14‐day observation period, individuals with regular cannabis use reported cannabis use on an average of 8.4 ± 2.5 days.

### 2.3 Ethical approvals

All participants signed a consent form approved by the Institutional Review board at Oregon Health & Science University and the study was pre‐registered on clinicaltrials.gov (NCT03560934). All methods were conducted in accordance with the relevant guidelines contained within the Declaration of Helsinki and regulations for human subjects protection. All participants provided written informed consent prior to participation in the study procedures and were informed they could withdraw from the study at any time.

### 2.4 Study procedures

Supine baseline blood pressures were sampled at 3, 8, 13, and 18 min. During the 30‐min tilt test, blood pressures were sampled 1 min after the start of tilt up and every 3 min thereafter. ECG was continuously monitored for heart rate and arrhythmias, and self‐rated (range 0–10) symptoms of nausea, dizziness, discomfort, or other abnormal sensations were surveyed after every blood pressure recording. Tests were aborted whenever any of the following signs and/or symptoms appeared (criteria for pre‐syncope): (1) Sustained low systolic blood pressure of <80 mmHg (detected via sphygmomanometer), or 15 mmHg below baseline (excluding cases with stable systolic blood pressure >100 mmHg); (2) Heart rate decrease >20 bpm from baseline (bradycardia) or asystole for ≥5 s; (3) Other symptoms of imminent syncope, including feeling faint, nauseous, tunnel vision/blacking out, and being unresponsive to questions, or participants' request of being tilted down due to these symptoms. In case of presyncope, the table was lowered to the horizontal position and the participant maintained the supine posture until symptoms resolved and/or cardiovascular parameters returned to baseline. ECG recordings were imported into an analysis software (Labchart 8 Pro, AdInstruments, Sydney, Australia) for heart rate variability (HRV) analysis. ECG recordings of sufficient quality were obtained in 8 individuals from the no cannabis use group the morning after placebo, and 7 individuals the morning after THC administration. ECG recordings of sufficient quality were obtained in 6 individuals from the regular cannabis use group the morning after placebo and 7 individuals the morning after THC administration. Time domain HRV was expressed by the percentage of R‐R intervals that varied by 50 ms or more (pNN50), the standard deviation of the R‐R interval of normal sinus beats (SDNN), and the root mean square of successive R‐R interval differences (RMSSD). The integrated area within the high‐frequency (HF‐HRV; 0.15–0.4 Hz) and low‐frequency (LF‐HRV; 0.04–0.15 Hz) ranges was obtained and we calculated normalized high frequency power by dividing integrated high frequency spectra by the total power (LF + HF) and multiplying by 100.

All fluid intakes including water (the only beverage provided), IV flushes or IV saline, and outs (i.e., urine volume) were measured throughout the study. An IV catheter was placed for regular blood draws as part of the parent study.

### 2.5 Data analysis

To minimize the influence of postural transition and anticipatory responses to the tilt procedure, blood pressure measurements taken during supine rest at minutes 8 and 13 were averaged to derive baseline blood pressure values. Similarly, HRV metrics were calculated as the average from the last 5 min of the supine rest period and used as baseline values. HRV metrics from the first 5 min of head‐up tilt and blood pressure values from the first 10 min (sampled at minutes 3, 6, and 9) were averaged and used to quantify cardiovascular reactivity to tilt. HRV metrics from the last 5 min and blood pressures from the last 10 min of supine recovery were used as the recovery time point. One individual in the no cannabis use group during the placebo treatment only had a blood pressure at min 3 due to early tilt termination. To calculate cardiovascular reactivity to tilt between groups and treatment conditions, we calculated changes from baseline to tilt for blood pressure values and heart rate. Fluid balance was calculated by taking the difference between total fluid intake and urine output 24 h leading up to the placebo and THC trials.

### 2.6 Statistical analysis

For statistical comparisons, we used a mixed model fit using restricted maximum likelihood (GraphPad Prism 11, Graphpad Software Inc., La Jolla, CA) with treatment (placebo or dronabinol), Time (Rest, Tilt, and Recovery) and group (cannabis use or no cannabis use) as fixed effects and participant as a random factor to assess cardiovascular variables during baseline, tilt, and recovery. If a significant treatment × group × time effect was detected, fishers LSD post‐hoc comparisons were performed. Mixed effect models were similarly used to assess cardiovascular reactivity and fluid balance with treatment and group as fixed effect and participant as a random factor. If a significant treatment × group effect was detected, fishers LSD post‐hoc comparisons were performed. Statistical significance was set at *p* ≤ 0.05.

## 3 RESULTS

Heart rate, heart rate variability, and arterial pressures the mornings after placebo administration and THC administration for individuals with no cannabis use and those who regularly use cannabis at baseline, tilt, and recovery are presented in Table 1. A significant interaction (*p*‐value group × treatment × time = 0.03) was detected for diastolic arterial pressure, with post‐hoc tests indicating that within the no cannabis use group, diastolic blood pressure was reduced during tilt the morning after THC consumption compared to tilt the morning after placebo. Values for plasma Δ^9^‐THC levels across the night and upon awakening for this sample population were previously reported (Gonzalez et al., 2026).

**TABLE 1:** Cardiovascular variables at rest, tilt, and recovery with mixed‐effects model results.

Variable	Time	No cannabis use (mean ± SD)		Regular cannabis use (mean ± SD)		*p*‐value (mixed effects model)						
		Placebo	Δ^9^‐THC	Placebo	Δ^9^‐THC	Group	Treatment	Time	Group × treatment	Group × time	Treatment × time	Group × treatment × time
Heart rate, bpm	Baseline	62.2 ± 11.8	66.8 ± 7.3	68.7 ± 7.9	66.4 ± 6.5	0.49	0.99	<0.01	0.18	0.78	0.17	0.08
	Head‐up tilt	77.3 ± 9.7	69.3 ± 11.9	74.4 ± 9.3	73.7 ± 7.6							
	Recovery	63.3 ± 12.5	68.7 ± 9.9	69.5 ± 7.9	68.9 ± 7.2							
SDNN, ms	Baseline	76.8 ± 22.2	96.9 ± 37.7	81.6 ± 32.0	79.6 ± 35.1	0.88	0.31	<0.01	0.54	0.44	0.54	0.06
	Head‐up tilt	55.9 ± 17.1	60.4 ± 41.4	56.0 ± 15.5	67.9 ± 20.7							
	Recovery	87.4 ± 38.1	64.9 ± 17.4	77.0 ± 16.3	93.4 ± 34.7							
RMSSD, ms	Baseline	57.6 ± 24.5	84.2 ± 63.8	48.0 ± 17.2	61.9 ± 57.3	0.65	0.15	<0.01	0.69	0.60	0.68	0.18
	Head‐up tilt	20.5 ± 8.5	43.7 ± 74.7	25.3 ± 13.4	34.1 ± 34.8							
	Recovery	66.0 ± 41.3	44.6 ± 15.6	43.7 ± 12.0	72.9 ± 68.1							
PNN50, %	Baseline	30.8 ± 18.0	25.1 ± 13.1	25.5 ± 16.1	18.6 ± 3.6	0.33	0.08	<0.01	0.81	0.40	0.20	0.57
	Head‐up tilt	3.9 ± 3.8	2.7 ± 3.5	3.3 ± 5.3	3.5 ± 3.6							
	Recovery	35.5 ± 21.5	22.7 ± 14.6	23.2 ± 13.4	18.9 ± 8.4							
Normalized high frequency, arb	Baseline	38.4 ± 14.9	43.1 ± 22.9	41.8 ± 12.8	32.8 ± 12.6	0.52	0.75	<0.01	0.42	0.88	0.51	0.31
	Head‐up tilt	12.6 ± 8.3	19.6 ± 17.6	15.5 ± 7.3	15.5 ± 10.1							
	Recovery	37.6 ± 18.3	39.6 ± 21.6	33.0 ± 14.4	39.8 ± 13.0							
SAP, mmHg	Baseline	112.4 ± 9.2	117.0 ± 12.2	117.0 ± 12.2	113.9 ± 7.8	0.83	0.80	0.35	0.60	0.44	0.47	0.14
	Head‐up tilt	112.2 ± 11.0	112.4 ± 14.5	114.9 ± 13.3	114.1 ± 12.1							
	Recovery	113.8 ± 10.3	117.1 ± 13.7	113.8 ± 11.6	114.9 ± 7.8							
DAP, mmHg	Baseline	64.6 ± 9.5	66.8 ± 7.4	68.8 ± 7.9	66.4 ± 6.5	0.48	0.21	<0.01	0.75	0.88	0.11	0.03
	Head‐up tilt	76.0 ± 6.1	69.3 ± 11.9^a^	74.4 ± 9.3	73.7 ± 7.6							
	Recovery	66.8 ± 9.0	69.1 ± 10.2	69.1 ± 7.6	69.0 ± 8.5							

*Note*: *p*‐values in each row represent overall fixed effects from a single mixed‐effects model including group, treatment, time (supine rest, head‐up tilt, recovery), and their interactions. Means are shown separately for each phase for descriptive purposes (means ± SD).

^a^ Indicates significant difference within group for matched time point. Heart rate, systolic arterial pressure (SAP), diastolic arterial pressure (DAP), the standard deviation of the R‐R interval of normal sinus beats (SDNN), the root mean square of successive R‐R interval differences (RMSSD)and the percentage of R‐R intervals that varied by 50 ms or more (pNN50). Heart rate, SAP, and DAP for no cannabis use group morning after placebo (*N* = 9) and morning after THC (*N* = 8); Regular cannabis use group morning after placebo and THC (*N* = 7). SDNN, RMSSD, PNN50, and normalized high frequency HRV metric for no cannabis use group morning after placebo (*N* = 8) and morning after THC (*N* = 7); Regular use group morning after placebo (*N* = 6) and morning after THC (*N* = 7).

In the placebo condition, one individual from the no cannabis use group requested an early tilt termination before blood pressure or symptoms were measured. Blood pressure values on one individual with no cannabis use the morning after THC administration are not included because they ended the study before the scheduled tilt table test. Lastly, in the regular cannabis use group, one individual did not consent to the tilt table test, and one individual was excluded from hemodynamic analysis due to the presence of an arrythmia. Subjective tilt table responses were collected in all available participants for both morning after placebo (no cannabis use, *N* = 9; regular cannabis use, *N* = 8) and morning after THC dosing (no cannabis use, *N* = 8; regular cannabis use, *N* = 8) conditions. No participants in the cannabis use group terminated the tilt test early following placebo or THC administration. Comparatively, in the no cannabis use group, 2 out of 8 individuals terminated the test early due to nausea/lightheadedness the morning after THC administration. Reports of dizziness were also more frequent in the no cannabis use group following THC (6/8) compared to placebo (3/9), whereas only (3/8) individuals who regularly use cannabis reported dizziness following both placebo and THC administration.

**FIGURE 1:**
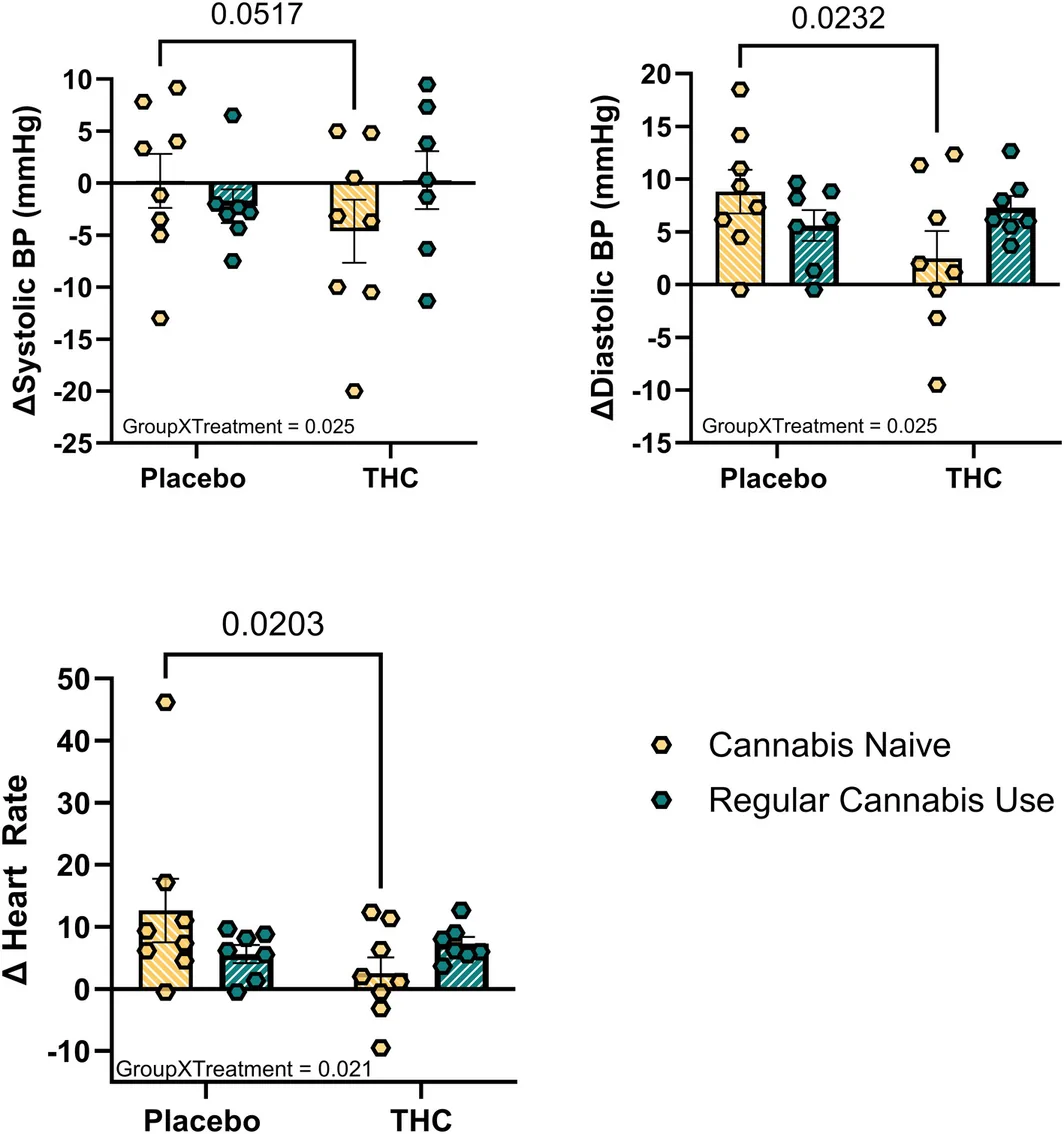
Blood pressure and heart rate reactivity to tilt. Changes in systolic arterial pressure, diastolic arterial pressure, and heart rate during tilt from baseline represented as individual data points and bar graphs. Changes in systolic arterial pressure, diastolic arterial pressure, and heart rate during tilt from baseline represented as individual data points and bar graphs. No cannabis use placebo (*n* = 8) due to missing blood pressure value. No cannabis use‐THC (*n* = 8) due to participant ending study early. Regular cannabis use placebo and THC (*n* = 7) due to one participant not consenting to test and one participant presenting with arrhythmia. Mean ± SD.

**FIGURE 2:**
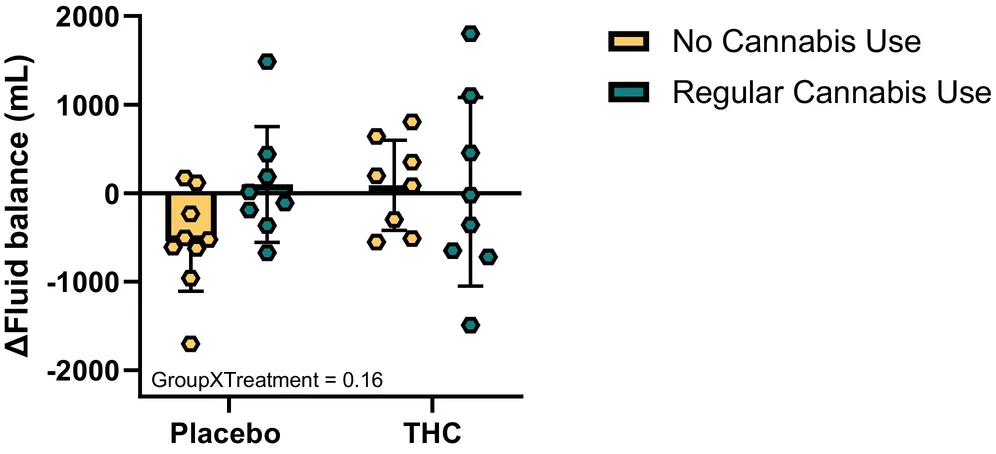
Fluid balance. Fluid balance calculated by the difference between total fluid intake and urine output preceding the tilt table test. Outcomes are shown as individual data points overlaid on bar graphs. Sample sizes vary by group due to incomplete data: the no cannabis use–placebo group includes *n* = 9, and the no cannabis use–THC group includes *n* = 8 due to one participant ending the study prior to testing; the regular cannabis use placebo and THC groups include *n* = 8 due to one participant not consenting to perform the tilt table test. Means ± SD.

The morning after THC administration, individuals with no cannabis use exhibited a significant reduction in both diastolic blood pressure (*p*‐value Group × Treatment = 0.02) and heart rate reactivity (*p*‐value Group × Treatment = 0.02) to tilt compared to the morning after placebo administration (Figure 1). Additionally, systolic arterial pressure reactivity to tilt was numerically reduced but not statistically significant (*p* = 0.052) in individuals with no cannabis use the morning after THC administration. Lastly, by calculating total water intake and total urine output, no statistically significant differences in fluid balance were detected in either group leading up to tilt table testing (*p*‐value Group × Treatment = 0.16; Figure 2).

## 4 DISCUSSION

The present ancillary analysis of a parent study assessed the cardiovascular responses to an orthostatic stress, the morning after placebo administration and the morning after 10 mg THC administration in both individuals with no cannabis use and individuals who regularly use cannabis. Our findings indicate that the morning after bedtime THC ingestion, individuals with no cannabis use exhibited modified hemodynamic responses to an orthostatic stress. In individuals with no cannabis use, diastolic blood pressure was attenuated during tilt the morning after THC consumption compared to tilt the morning after placebo. Exposure to an orthostatic stress the morning after THC ingestion resulted in reduced heart rate and diastolic blood pressure reactivity in individuals with no cannabis use despite similar hydration between trials. Additionally, the morning after THC ingestion, 6 out of 8 individuals with no cannabis use reported sensations of dizziness (compared to 3 out of 9 the morning after placebo) and 2 reported severe nausea resulting in tilt test termination (compared to zero the morning after placebo). Our results suggest that before bedtime THC ingestion in individuals with no cannabis use may impair next morning orthostatic tolerance.

The use of cannabis products as sleep aids is increasing despite inadequate evidence of their efficacy or safety. In the United States and Canada, the prevalence of sleep aid use has more than doubled over the last two decades (Reuben et al., 2023) with 15.6% of Canadians using cannabis‐derived products for sleep aids (Morin et al., 2024). There is currently little evidence to suggest clinically significant next‐morning (“hangover”) physiological effects associated with evening cannabis use (Chait, 1990). Previous investigations have reported impaired memory and increased sleepiness the morning after administration of 15 mg of THC (oromucosal spray) (Nicholson et al., 2004). However, oral THC ingestion and higher doses of THC are readily available in legal markets and may increase the likelihood of next‐morning physiological effects following bedtime use (Cousens & DiMascio, 1973; Hasin et al., 2023). Mild to moderate feelings of being “hungover,” have been reported after orally administered doses of 30 mg of THC (Cousens & DiMascio, 1973). Our study is the first to report next‐morning cardiovascular responses following bedtime THC ingestion: primarily, the blunting of blood pressure and heart rate reactivity to orthostatic stress in participants with no cannabis use history. Impaired orthostatic control of blood pressure is associated with an increased risk of falls, especially in older adults (Shaw et al., 2015). From 2013 to 2019, cannabis use has more than doubled among older adults (50–64 and >65 years) (Mattingly et al., 2024), and these individuals regularly use cannabis for health reasons, including as therapy for poor sleep (Haug et al., 2017), often without medical guidance (Baumbusch & Sloan, 2021). Given our results in healthy young adults, further investigation is warranted in older adults as it may increase orthostatic instability and fall risk in this group.

Within 2–3 min of assuming head‐up tilt, about 10% of total blood volume is redistributed caudally, triggering compensatory reflexes coordinated by the autonomic nervous system to maintain hemodynamic stability. The most crucial compensatory mechanisms for maintaining hemodynamic stability during an orthostatic challenge are sympathetically mediated increases in peripheral resistance and vagal withdrawal at the heart, allowing for cardiac acceleration, both of which are coordinated by the baroreflex (Cheshire & Goldstein, 2019). THC is known to acutely affect multiple mechanisms under autonomic control, such as vascular smooth muscle (Weiss et al., 1972), vagal tone (Pabon et al., 2022), and sympathetic outflow (Cheung et al., 2022). Early investigations by Weiss et al. on the effects of THC on cardiovascular variables reported that intravenous infusion of THC increased heart rate, blood pressure, and forearm blood flow, with reduced reflexive vasoconstriction to a deep breath (Weiss et al., 1972). Additionally, upon exposure to tilt, blood pressure transiently decreased, resulting in presyncope in some participants. However, there was no obvious explanation for the transient decrease in blood pressure during tilt, as participants did not exhibit impaired vasoconstriction (Weiss et al., 1972). Acute cannabis and acute THC use are also known to induce postural dizziness upon standing, with symptoms peaking immediately following drug use (Mathew et al., 2003). Individuals who regularly use cannabis may develop tolerance to its psychological and physiological effects, as repeated exposure attenuates the tachycardic response to THC (Colizzi & Bhattacharyya, 2018). Therefore, it is plausible that individuals who regularly use cannabis may have reduced likelihood of experiencing orthostatic instability and the associated symptoms the morning after THC ingestion. The participants in this study abstained from all caffeine sources leading up to and during the in‐laboratory stay. In real‐world settings, many individuals routinely consume caffeine in the morning, which may offset blunted orthostatic responses (Gibbon & Frith, 2021). The results of our study are primarily descriptive in nature and do not provide mechanistic insight into how THC may impair orthostatic tolerance or why we did not see similar results in individuals who regularly use cannabis. Future investigations should combine tilt table testing with other autonomic measures and tests, such as beat‐to‐beat arterial pressure and microneurography, along with the Valsalva maneuver, to distinguish if acute THC ingestion impairs cardiovagal or sympathetic baroreflex sensitivity. Although no statistically significant differences were detected in HRV metrics, trends in several measures warrant investigation in a larger sample size. Lastly, more studies are needed to determine if regular cannabis use results in physiological adaptations to the effects of THC.

Our study has several strengths such as controlled nutritional intake in the laboratory environment, and standardized participant routines while in the laboratory allowing us to conduct the tilt protocol at a routine time of day. Despite the rigorous design of this study, there are several limitations to consider. Our sample size was limited, which may have prevented detecting differences in blood pressure reactivity and heart rate variability between groups. THC dosing in this study was controlled and in real life situations THC dosages may vary widely, as THC is not FDA regulated. Our study lacked beat‐to‐beat assessment of blood pressure and thus we are unable to comment on real‐time hemodynamic decompensation to tilt or assess baroreflex impairment. Hydration status should be formally assessed in future studies. Lastly, this was a single blind study and oral THC administration was not randomized, and we cannot rule out the possibility of an order effect interacting with the drug or group effect in our analyses.

In conclusion, bedtime THC ingestions do not alter morning resting cardiovascular variables in either individuals with no cannabis use or in individuals who regularly use cannabis. However, our ancillary findings suggest that bedtime THC ingestion impairs morning blood pressure and heart rate reactivity to an orthostatic challenge in individuals with no cannabis use but not in individuals who regularly use cannabis. Additionally, there was an observed increase in dizziness and nausea among the individuals with no cannabis use during tilt the morning after THC ingestion. Impaired orthostatic stability the morning after THC use may have implications for individuals considering using THC products as sleep aids.

## AUTHOR CONTRIBUTIONS

**Joshua E. Gonzalez:** Data curation; formal analysis; funding acquisition; investigation; project administration; resources; supervision; validation; visualization. **Alicia V. Stewart:** Data curation; formal analysis; investigation; project administration. **LaTroy D. Robinson:** Data curation; formal analysis; project administration. **Noal A. Clemons:** Data curation; formal analysis; project administration; resources. **Omar H. Ordaz:** Data curation; formal analysis; project administration. **Jonathan S. Emens:** Investigation; project administration; supervision. **Saurabh S. Thosar:** Investigation; project administration; supervision. **Steven A. Shea:** Conceptualization; funding acquisition; investigation; project administration; supervision. **Nicole P. Bowles:** Conceptualization; data curation; formal analysis; funding acquisition; investigation; methodology; project administration; resources; supervision; validation; visualization.

## FUNDING INFORMATION

This work was supported by a Focused Projects Award from the American Academy of Sleep Medicine (to N.P. Bowles) and the National Institutes of Health Grants K01 HL151745 (to N.P. Bowles), R35 HL155681 (to S.A. Shea), T32 HL083808 (to J.E. Gonzalez) and the Oregon Institute of Occupational Health Sciences at Oregon Health & Science University via funds from the Division of Consumer and Business Services of the State of Oregon (ORS 656.630).

## Data Availability

Data supporting the conclusions of this manuscript will be made available by the corresponding author, upon request, to any qualified researcher.
